# Supplementary material for: Decoupling between SARS-CoV-2 transmissibility and population mobility associated with increasing immunity from vaccination and infection in South America
Source: Sci Rep. 2022 Apr 27;12:6874. doi: 10.1038/s41598-022-10896-4 (PMC9044384; doi:10.1038/s41598-022-10896-4)
Supplement: Supplementary file 1 — Supplementary Information. [file 41598_2022_10896_MOESM1_ESM.pdf]

Supplementary Material
Decoupling between SARS-CoV-2 transmissibility and population mobility associated with increasing immunity from vaccination and infection in South America

Marcelo Fiori*† Gonzalo Bello‡ Nicolás Wschebor§ Federico Lecumberry¶
Andrés Ferragut‖ Ernesto Mordecki**

A Supplementary Material

A.1 Additional countries

In figures A.1 and A.2 we provide the same analysis shown in figures 1 and 2 respectively, for the case of Israel and Italy. It can be observed that the same phenomenon described in the main text takes place in these countries. Namely, a first period where the mobility successfully fits and predicts the reproductive number R_t, and after the effect of the vaccination starts to complement the immunity from natural infection, a period where the R_t decouples from the mobility.

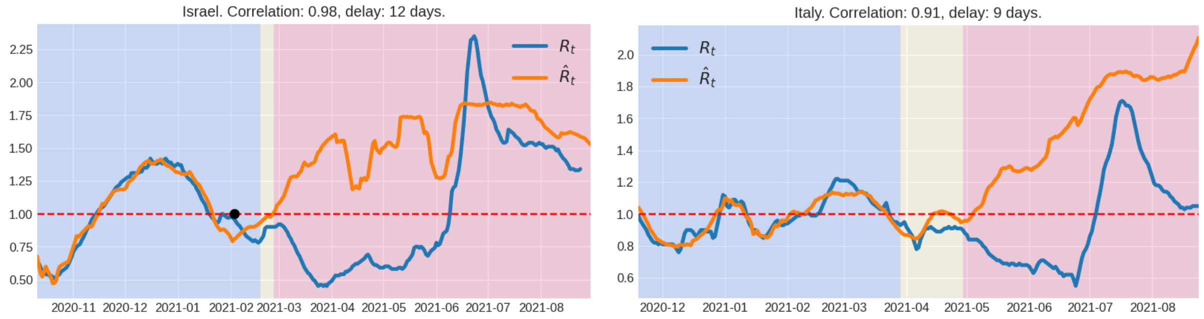

Figure A.1: Viral effective reproduction number R_t and its estimation $\hat{R}_t$ using mobility information. Background colors indicate the following time periods: in blue, the time period used to fit the linear model (see Section 4.2), in yellow, the period after the fitting, but before the decoupling point, and in red after the decoupling point. The black dot corresponds to the last time the reproductive number was above one. The correlation corresponds to the period used to fit the model. The delay indicated is the time-shift between the mobility time series and R_t in order to maximize the correlation in the linear regression.

*Corresponding author: mfiori@fing.edu.uy
†Instituto de Matemática y Estadística "Rafael Laguardia", Facultad de Ingeniería, Universidad de la República, Uruguay
‡Instituto Oswaldo Cruz (FIOCRUZ), Rio de Janeiro , Brazil
§Instituto de Física, Facultad de Ingeniería, Universidad de la República, Uruguay
¶Instituto de Ingeniería Eléctrica, Facultad de Ingeniería, Universidad de la República, Uruguay
‖Facultad de Ingeniería, Universidad ORT, Uruguay
**Centro de Matemática, Facultad de Ciencias, Universidad de la República, Uruguay

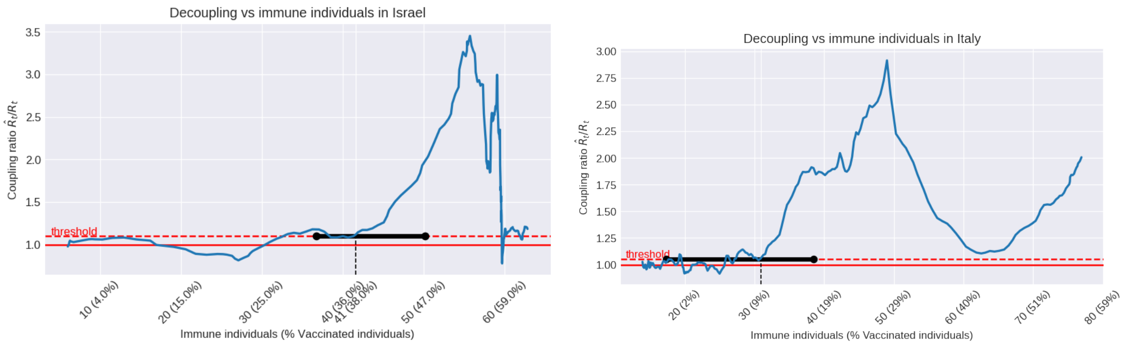

Figure A.2: Coupling ratio $\hat{R}_t/R_t$ plotted with respect to the percentage of immune population. During the first months of 2021 the coupling ratio varies around 1, which corresponds to the periods where the R_t and $\hat{R}_t$ are in concordance in Figure A.1. Immune population includes immunity achieved by vaccination (taking into account its effectiveness), and natural infection (see subsection 4.3). The percentage of people fully vaccinated is described as well.

A.2 Further methodological details and validation

A.2.1 Estimating the reproduction rate

For the analysis in this paper we considered several possible estimations of the time-varying reproduction rate R_t of the epidemic. We now briefly review the available approaches and explain our decision to settle on a given estimator for R_t.

A first approach, proposed in [3], consists on estimating R_t directly from incidence data I_t, the number of observed new cases. The estimation proceeds as follows: assume that when a person becomes infected, it can start spawning new infections from its contacts. These new infections will be reflected in the number of cases (become detected) after a *random* period of time, which can be modeled by a probability distribution w_s on $s > 0$. This is called the *serial interval* of the disease, and for SARS-CoV-2 it has been estimated by [4] as having a mean of 3.95 days and a standard deviation of 4.75 days. For definiteness we assume a discrete Gamma distribution for $\{w_s\}$ with these mean and variance.

Assuming that the number of contagions generated by an individual is Poisson and independent across individuals, the number of new infections at time t follows also a Poisson distribution given by:

$$I_t \sim \text{Poisson}\left(R_t \Lambda_t\right), \tag{1}$$

where R_t is the current reproduction rate that we wish to estimate, and Λ_t is given by:

$$\Lambda_t = \sum_{s>0} w_s I_{t-s}. \tag{2}$$

The intuition behind Λ_t is that this should be the average number of new infections reported at time t for a reproduction rate of 1.

The authors of [3] then propose to use a Bayesian approach. Assuming R_t is approximately constant over a window of length τ, and that a priori is distributed as a Gamma random variable with shape parameter a and scale parameter b, the a posteriori distribution of R_t can be computed and a suitable estimation of R_t is obtained as:

$$R_t = \frac{a + \sum_{k=t-\tau+1}^{t} I_k}{\frac{1}{b} + \sum_{k=t-\tau+1}^{t} \Lambda_k} \tag{3}$$

Eq. (3) has a simple intuitive explanation: besides a small bias from the a priori parameters, it is the ratio between the new observed cases in a given time window to the number of expected cases for a reproduction rate of 1.

The main advantage of this method is that it makes little assumptions on the dynamics of the epidemic, only dealing with disease specific parameters and the reasonable Poisson assumption on contacts. The main disadvantage is that, in order to be robust against the noise in observations and cope with weekly seasonal effects observed in the data, we have to employ a pretty large estimating window τ (typically between 7 and 14 days). This introduces a significant *lag* in the estimation. With data up to time t, we are estimating the value of R_t with a delay of up to 1 week. Since we are interested in the time correlations between mobility and the *current value* of the reproduction rate, this lag precludes us from using this robust estimator.

A second approach is proposed in [5], and is currently computed in real time for a list of countries in [1]. The authors assume a simple SIR model for the dynamics of currently active cases, which we denote by A_t to avoid using the standard name I since we reserve I for *incidence* or *new infections*.

The dynamics of the active cases follow the evolution equation:

$$A_t = A_{t-1} + \gamma\,(R_{t-1} - 1)\,A_t. \tag{4}$$

Here $1/\gamma$ is the recovery rate, i.e. the average time a person stops spreading the disease. In the typical SIR model, $R_t = \beta_t S_t(N)$, where β_t is the current level of social interaction and S_t/N the ratio of susceptible population. However, since we are interested in quantifying only the reproduction rate, we can employ directly eq. (4).

A simple transformation of (4) expresses the *growth rate* of the active cases as a function of R_t:

$$gr(A_t) := \frac{A_t - A_{t-1}}{A_{t-1}} = \gamma(R_{t-1} - 1). \tag{5}$$

Moreover, for small relative increments this can be further simplified using the approximation $\log(1+x) \approx x$ to write:

$$\nabla \log(A_t) = \gamma(R_{t-1} - 1), \tag{6}$$

where ∇ is the usual difference operator.

Since most of the data available is for *incidence* of new cases I_t, in order to construct the time series A_t one resorts again to the SIR model equations to write:

$$A_t = (1-\gamma)A_{t-1} + I_t. \tag{7}$$

The complete procedure is as follows: given a time series data from case counts I_t, construct the series of active cases using (7). Then model the growth rate evolution of A_t by using a simple *local level model* [6] for the trend in the reproduction rate R_t given by:

$$\nabla \log A_t = \gamma(R_{t-1} - 1) + \varepsilon_t, \tag{8a}$$

$$R_t = R_{t-1} + \eta_t. \tag{8b}$$

where ε_t, η_t are measurement noises assumed Gaussian. A suitable estimation of R_t can be directly obtained from eqs. (8) by applying the Kalman filtering technique [6]. The main advantages of this approach are two-fold: first they are extremely robust to noise in the measurements I_t and consequently A_t. The second advantage is that the estimate is real-time, i.e. it introduces no lag on the trend. This is perfectly suited for our purposes where we want to characterize the time correlation with the mobility estimation.

During preparation of this manuscript, we enhanced the model in eqs. (8) to include cyclic components in order to model systematic weekly trends in the data. However, inclusion of these trends did not change significantly the estimated trend of the R_t trajectory, so for all our analysis, we settled on the local level model approach and Kalman filtering of (8).

A.2.2 Backtesting/Validation of regression

Although the regression of the mobility features to the R_t is fitted in the period with blue background in Figure 1, and therefore period with yellow background serves as a validation of this regression up to the decoupling time, in this section we provide further details and validation.

For all five countries, we took the period prior to the decoupling time, and divided it into two sections of approximately the same length. The first period is used to fit the regression parameters, and the second period is used to validate the fitting. Additionally, we compute the variance of the regression parameters in the standard way, and use them to draw Monte Carlo trajectories in order to build error bands around the regression curves. The results are shown in Figure A.3, where the correlation in both the training and validation period are also reported. The results tend to reasonably validate the obtained the regression.

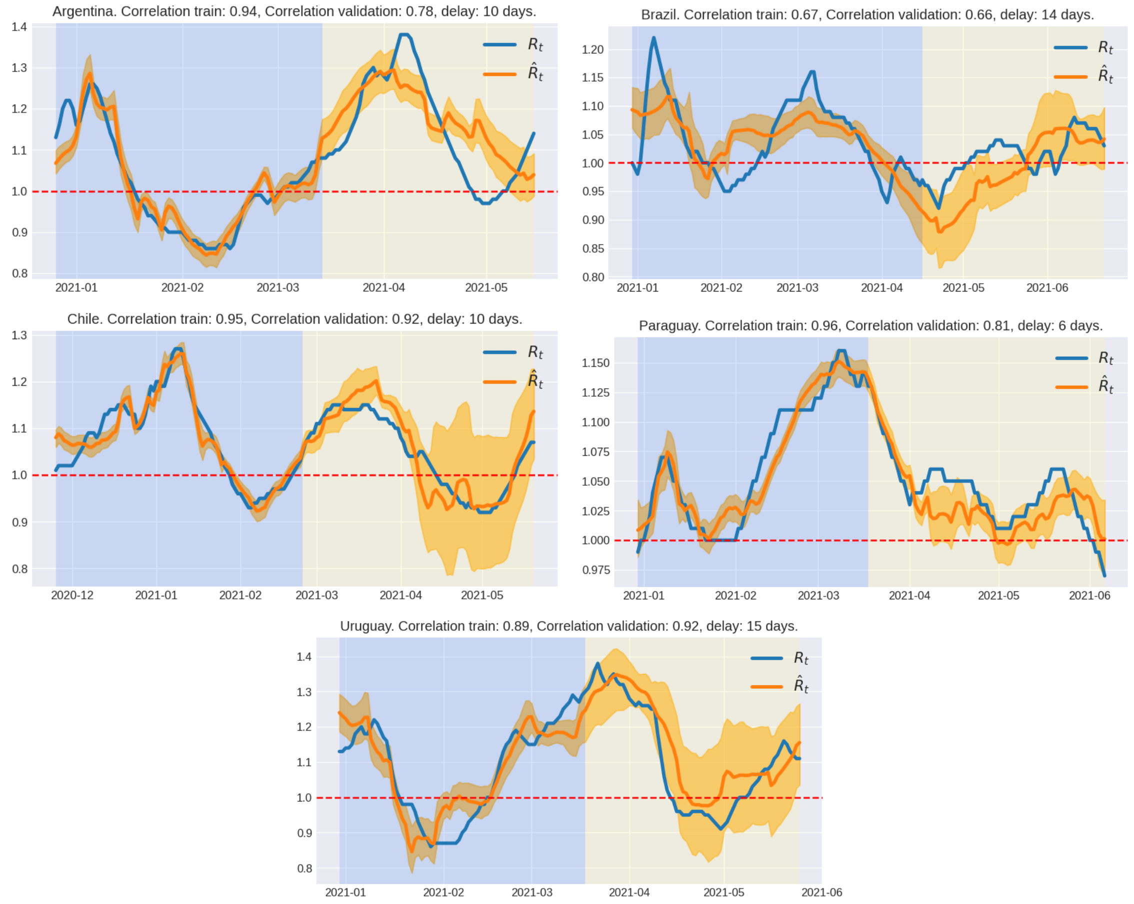

Figure A.3: Backtesting/validation of regression. The period with blue bakcgound is used to compute the regression, and the period with yellow background is used to validate the fitting. Error bands computed by Monte Carlo simulations, using the covariance of the regression parameters. The correlation in both the training an validation periods are reported in each panel.

A.2.3 Sensitivity to the threshold

As described in the main text, the decoupling time T_D was defined as the moment when the coupling ratio $\hat{R}_t/R_t$ definitely exceeds the threshold 1.10. In order to study the sensitivity to the value of this threshold, as well as to validate the methodology, we expose two additional experiments.

First, we present the decoupling time T_D and the corresponding immunity cHIT for several values of the threshold. It can be observed in Table 1 that the obtained cHIT values fall inside the confidence intervals in Table 1, and actually they are are very close to the central cHIT values presented.

Second, we present a decoupling time detection by means of an alternative method. Figure A.4 shows the coupling ratio $\hat{R}_t/R_t$, as well as the detected decoupling time D_t detected by the threshold (in dashed

Country	CPD	Threshold	T_D	cHIT
Argentina	May. 29	1.05	May. 30	29 %
		1.10	Jun. 02	29 %
		1.15	Jun. 03	30 %
		1.20	Jun. 06	33 %
Brazil	Jun. 22	1.05	Jun. 19	44 %
		1.10	Jun. 23	45 %
		1.15	Jun. 24	45 %
		1.20	Jun. 25	46 %
Chile	May. 28	1.05	May. 18	42 %
		1.10	May. 22	43 %
		1.15	May. 26	44 %
		1.20	May. 30	45 %
Paraguay	Jun. 12	1.05	Jun. 09	33 %
		1.10	Jun. 11	36 %
		1.15	Jun. 18	37 %
		1.20	Jun. 26	38 %
Uruguay	Jun. 10	1.05	May. 27	33 %
		1.10	May. 29	33 %
		1.15	Jun. 01	34 %
		1.20	Jun. 13	36 %

Table 1: Decoupling time (T_D) and cHIT (conditional herd immunity threshold) for different threshold values. The decoupling time detected by the Change Point Detection method (CPD) is also reported for comparison.

red), and the result of a standard Change Point Detection (CPD) algorithm [2] (in blue). It can be observed that both methods yield compatible results in all five countries.

References

[1] Ritchie H. et al. Coronavirus Pandemic (COVID-19), (2020). Published online at OurWorldInData.org. Retrieved from: https://ourworldindata.org/coronavirus. Accessed 2021-08-28.

[2] Killick, R., Fearnhead, P., and Eckley, I. A. Optimal Detection of Changepoints With a Linear Computational Cost. *Journal of the American Statistical Association*, 107, 1590–1598 (2012).

[3] Cori, A., Ferguson, N., Fraser, C. and Cauchemez, S. A New Framework and Software to Estimate Time-Varying Reproduction Numbers During Epidemics. *American Journal of Epidemiology*, 178(9), 1505–1512 (2013).

[4] Du Z, Xu X, Wu Y, Wang L, Cowling BJ, Meyers LA. Serial Interval of COVID-19 among Publicly Reported Confirmed Cases. *Emergent Infectious Diseases*, 26(6), 1341-1343 (2020).

[5] Arroyo-Marioli, F., Bullano, F., Kucinskas, S. and Rondón-Moreno, C. Tracking R of COVID-19: A new real-time estimation using the Kalman filter. *PLOS ONE*, 16, 1–16 (2021).

[6] Shumway, R. H., and Stoffer, D. S. *Time series analysis and its applications: With R examples* Springer, New York (2006).

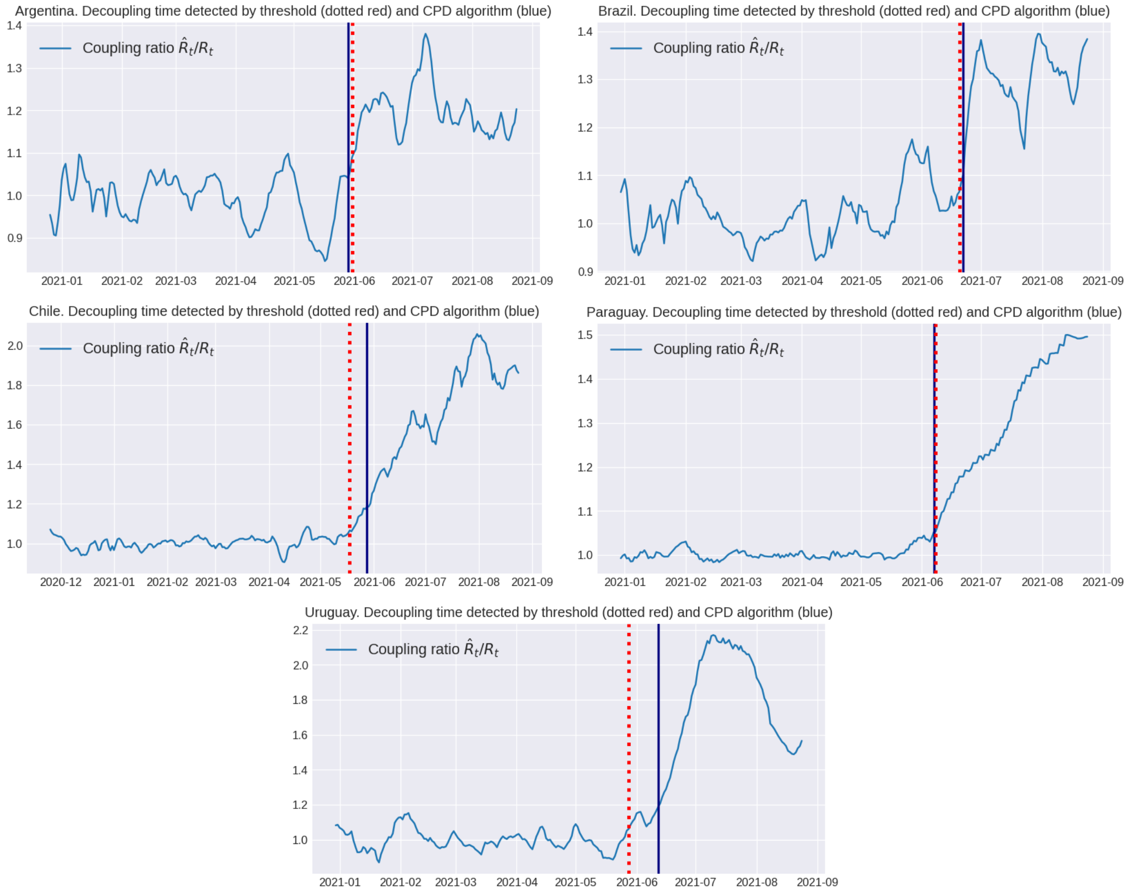

Figure A.4: Comparison of decoupling time detection as described in the main text (threshold), and by using a standard Change Point Detection method. Observe that the larger difference is only 12 days.
